# A prospective observational longitudinal study of new-onset seizures and newly diagnosed epilepsy in dogs

**DOI:** 10.1186/s12917-017-0966-y

**Published:** 2017-02-16

**Authors:** N. Fredsø, N. Toft, A. Sabers, M. Berendt

**Affiliations:** 1 Department of Veterinary Clinical and Animal Sciences, University of Copenhagen, Dyrlaegevej 16, 1870 Frederiksberg C, Denmark; 2 National Veterinary Institute, Section for Epidemiology, Technical University of Denmark, Frederiksberg C, Denmark; 3 The Epilepsy Clinic, Department of Neurology, University State Hospital (Rigshospitalet), Copenhagen, Denmark

**Keywords:** Canine epilepsy, Idiopathic epilepsy, Epileptic seizure recurrence, Epilepsy remission, Non-epileptic seizures

## Abstract

**Background:**

Seizures are common in dogs and can be caused by non-epileptic conditions or epilepsy. The clinical course of newly diagnosed epilepsy is sparsely documented.

The objective of this study was to prospectively investigate causes for seizures (epileptic and non-epileptic) in a cohort of dogs with new-onset untreated seizures, and for those dogs with newly diagnosed epilepsy to investigate epilepsy type, seizure type and the course of disease over time, including the risk of seizure recurrence. Untreated client-owned dogs experiencing new-onset seizures were prospectively enrolled in a longitudinal observational study including clinical investigations and long-term monitoring at the Copenhagen University Hospital for Companion Animals. A baseline clinical assessment was followed by investigator/owner contact every eight weeks from inclusion to death or end of study. Inclusion of dogs was conducted from November 2010 to September 2012, and the study terminated in June 2014.

**Results:**

One hundred and six dogs were included in the study. Seventy-nine dogs (74.5%) were diagnosed with epilepsy: 61 dogs (77.2%) with idiopathic epilepsy, 13 dogs (16.5%) with structural epilepsy and five dogs (6.3%) with suspected structural epilepsy. A non-epileptic cause for seizures was identified in 13 dogs and suspected in 10 dogs. Four dogs in which no cause for seizures was identified experienced only one seizure during the study. In dogs with idiopathic epilepsy 60% had their second epileptic seizure within three months of seizure onset. Twenty-six dogs with idiopathic epilepsy (43%) completed the study without receiving antiepileptic treatment. The natural course of idiopathic epilepsy (uninfluenced by drugs) was illustrated by highly individual and fluctuating seizure patterns, including long periods of remission. Cluster seizures motivated early treatment. In a few dogs with a high seizure frequency owners declined treatment against the investigators advice.

**Conclusions:**

Epilepsy is the most likely diagnosis in dogs presenting with new-onset seizures. The course of idiopathic epilepsy is highly individual and might not necessarily require long-term treatment. This must be considered when advising owners about what to expect with regard to treatment and prognosis.

## Background

Seizures in dogs can arise from non-epileptic causes and epilepsy. For owners of dogs with newly diagnosed epilepsy, most common concerns relate to the possible source of seizures, the risk of seizure recurrence, and prognosis.

Only few studies have investigated the distribution of seizures caused by epilepsy versus other conditions [1–3], whereas the risk of seizure recurrence in dogs with newly diagnosed and untreated epilepsy is unknown. Such information is of interest for the diagnostic and therapeutic approach to seizure patients. Furthermore information regarding seizure patterns in dogs with newly diagnosed untreated epilepsy is of special interest, as it might influence recommendations regarding when to start treatment.

For humans with untreated epilepsy, a 2-year recurrence risk of 40% has been estimated after a first unprovoked seizure [4–6].

It is a matter of controversy whether the risk of poor seizure control is increased if treatment is not initiated soon after diagnosis [7]. Studies in untreated people with epilepsy from resource poor countries have not found any association between remission rates and the duration of the epileptic condition or the total number of seizures prior to treatment [8, 9]. Other studies have however shown that the possibility of achieving long-term remission with treatment is higher for human patients treated after the first seizure compared to those treated after two or more seizures [10, 11]. Two human population studies, randomizing patients to immediate treatment or treatment only if a second seizure occurred, found that treatment after a first seizure reduced the short-term (1–2 years) risk of seizure recurrence, but found no difference between the two groups with respect to long-term remission [5, 6]. Overall, the risk of a second seizure was highest in the period shortly after the first seizure, and the risk of seizure recurrence was higher in patients, which had experienced two or more seizures at the time of diagnosis [4, 5]. Furthermore a higher risk of seizure recurrence has been documented in people with epilepsy caused by a structural abnormality [4, 12, 13].

Only few studies have provided information to support recommendations of when to initiate antiepileptic treatment in dogs. A recent study investigating 334 dogs with idiopathic epilepsy found that a high seizure density before initiating treatment influenced the likelihood of achieving remission with treatment negatively, while neither seizure frequency nor the total number of seizures prior to treatment influenced the likelihood of remission [14]. An older study including 54 Labrador Retrievers with idiopathic epilepsy reported a significantly better treatment response in dogs with a low seizure frequency and a low total number of seizures prior to treatment, compared to dogs with a high seizure frequency and a high total number of seizures prior to treatment [15].

Prospective longitudinal studies in dogs with new-onset seizures are desirable as they can serve to document the likelihood of a diagnosis of epilepsy and illustrate the course of epilepsy over time, including seizure recurrence patterns [16]. Such information can also be useful when trying to establish evidence-based recommendations regarding when to initiate therapy, and support clinicians when trying to advice owners of dogs with epilepsy about what to expect from the future.

The main aim of the present study was to investigate the causes and characteristics of seizures in dogs presented with new-onset seizures (epileptic or non-epileptic). For dogs diagnosed with epilepsy, it was furthermore an aim to report epilepsy type and associated seizure types and to document the natural course of disease over time, including seizure recurrence.

## Methods

### Study design, procedures and clinical assessment

The study was approved by the local institutional ethic committee at University of Copenhagen (2010-6/NAFM) and informed consent was obtained from the owners. The study was conducted as a prospective observational longitudinal study at the Copenhagen University Hospital for Companion Animals. The recruitment period lasted from November 2010 to September 2012, and the study was terminated in June 2014. Included dogs were monitored longitudinally including a baseline clinical assessment followed by prescheduled investigator-owner contacts every eight weeks (+/- two weeks). The dogs were followed from inclusion to death or end of study.

The study population consisted of client-owned dogs with a history of new-onset seizures. Only dogs which were drug naïve with respect to antiepileptic drugs (with the exception that prior treatment with diazepam for acute seizures was allowed) could be included in the study. Exclusion criterion was a first presentation of seizures as status epilepticus (SE).

Dogs were recruited from Danish first-line veterinary clinics and from the equally comparable first-line (community) Practice at the Copenhagen University Hospital for Companion Animals in the following way: Veterinarians were informed about the upcoming study through oral presentations and a mailed pamphlet describing the aim of the study, the inclusion and exclusion criteria and the clinical study protocol. Furthermore, the contact information for the primary investigator (NF) was detailed. The veterinarians were asked to support the study by sending client-owned dogs presenting with a history of new-onset seizures directly to the primary investigator (meaning that seizure work-up was then performed for all dogs by the primary investigator at the Copenhagen University Hospital for Companion Animals specialized Neurology Clinic). Dogs which fulfilled the above stated study criteria were included consecutively into the study by the primary investigator. All dogs included in the study underwent a standardized clinical work-up (a priori absolutely required diagnostic work-up) defined by the prospectively designed study protocol (see below) and all these clinical investigations were performed for each single dog by the primary investigator.

In this study a seizure was defined as: *“any sudden, short lasting and transient event and does not imply that the event is epilepsy” *[17]. Epilepsy was defined as: *“a disease of the brain characterized by an enduring predisposition to generate epileptic seizures. This definition is usually practically applied as having at least two unprovoked epileptic seizures > 24 h apart” *[17, 18].

Epileptic seizures were classified as primary generalized, focal or focal seizure evolving to become generalized. Post-ictal phase was defined as the time from end of clinical signs of seizure activity and until the dog regained its normal behavior. Cluster seizures were defined as > 1 epileptic seizure within 24 h. Cluster seizures were considered a single seizure event.

### Categorization of causes for seizures

**Fig. 1:**
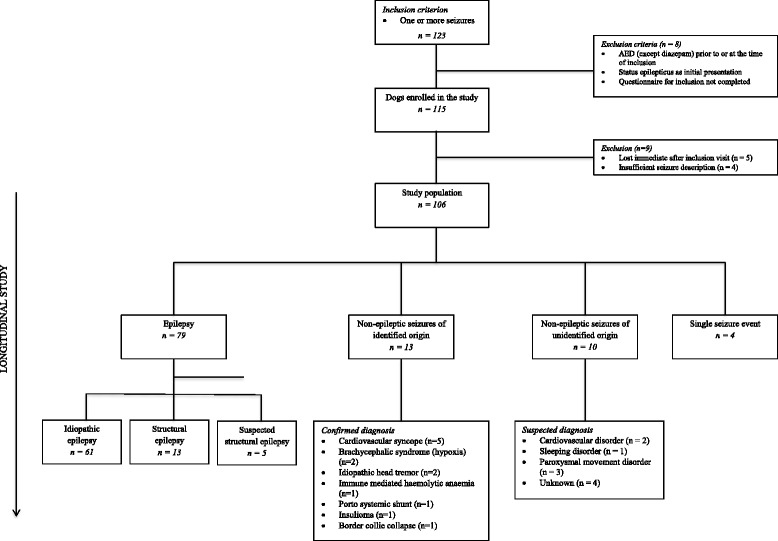
Flow diagram of the study

Dogs could be categorized into one of four diagnostic categories: 1) Epilepsy (idiopathic epilepsy, structural epilepsy, suspected structural epilepsy), 2) Non-epileptic seizures of identified origin, 3) Suspected non-epileptic seizures of unidentified origin and 4) Single seizure event (from inclusion to end of study) (Fig. 1).

A diagnosis of *idiopathic epilepsy* required two or more epileptic seizures of intracranial/cerebral origin occurring more than 24 h apart. Furthermore a normal interictal physical and neurological examination and blood profiles (complete blood count (CBC), biochemistry profile and thyroid profile (total thyroxin (TT4), free thyroxin (fT4) and thyroid stimulating hormone (TSH) within normal range (serving to secure that no other diseases known to cause seizures were present). The seizure semiology should be characterized by short-lasting convulsions occurring alone (generalized epileptic seizure), or by motor, autonomic or behavioral signs occurring alone (focal epileptic seizure) or followed by convulsions (focal epileptic seizure evolving to become generalized). Furthermore in the individual dog, seizure semiology should be identical from seizure to seizure. Furthermore dogs should from inclusion to death or end of study stay free of clinical signs of neurological disease indicating brain pathology (structural epilepsy or suspected structural epilepsy) or any indication of extracranial disease known to cause seizures and mimic epilepsy. Magnetic resonance imaging (MRI) of the brain was strongly recommended for all dogs, but owners could decline due to ethical reasons. Computerized Tomography (CT) and cerebrospinal fluid (CSF) analysis was recommended by indication.

A diagnosis of *structural epilepsy* required two or more epileptic seizures occurring more than 24 h apart where a structural intracranial/cerebral cause for seizures was identified by diagnostic imaging (MRI/Computer tomography (CT), CSF analysis, or post mortem findings alone or in combination.

In order to be categorized with *suspected structural epilepsy* two or more epileptic seizures occurring more than 24 h apart of suspected intracranial/cerebral origin should have occurred. Intracranial/cerebral pathology was thus strongly suspected but could not be confirmed, typically because the owners declined further work-up with MRI, CSF analysis and/or post mortem examination. This category included e.g. dogs with a medical history of previous head trauma or resolved cerebral disease, post-disease occurring seizures and no indication of other diseases known to mimic epilepsy, and dogs with interictal clinical lateralized or focal neurological signs (progressive or non-progressive) indicating localized cerebral brain pathology.

Dogs were categorized with *non-epileptic seizures of identified origin* if a non-epileptic cause of seizures was identified by relevant diagnostic tests, or in case a known breed specific episodic non-epileptic seizure-condition such as e.g. idiopathic head tremor in the English bulldog [19], was diagnosed.

Dogs were categorized with *suspected non-epileptic seizures of unidentified origin* in cases where the clinical appearance of seizures was atypical for epilepsy (e.g. long-lasting >10 min seizures or solely exercise induced seizure events) and where a cause for seizures could not be identified based on the available diagnostic work-up or because of incomplete work-up (typically if owners declined the suggested diagnostic tests).

### Baseline assessment

At inclusion a baseline clinical assessment (using the prospectively designed study protocol) was performed by the primary investigator (NF) at the Copenhagen University Hospital for Companion Animals specialized Neurology Clinic for all dogs participating in the study. All dogs were investigated clinically using a problem oriented medical record (POMR) approach and a differential diagnosis list based on the mnemonic acronym DAMNIT-V. Clinical investigations included the following: the dogs ID and signalment, full medical and seizure history, physical and neurological examination, CBC, biochemistry and thyroid profile. If a diagnosis other than epilepsy was suspected relevant diagnostic tests such as e.g. echocardiography, ultrasonography, radiographs and additional blood tests were introduced in order to reach a final (differential) diagnosis. For all dogs suspected of epilepsy MRI was strongly recommended. Other specialized neuro-diagnostic tests such as e.g. CT and CSF analysis were performed by indication.

Seizure history was obtained as a face to face interview using a structured questionnaire (Questionnaire A) addressing age at seizure onset, date of first seizure and date of subsequent seizures from first seizure to inclusion, seizure frequency including time for seizures to occur and possible occurrence of cluster seizures or status epilepticus. Furthermore information was collected regarding seizure semiology (focal and generalized phenomenology), duration of seizure(s), possible seizure triggering events, possible events preceding seizures, and postictal phase. Finally, information was collected regarding, possible complications at birth, previous head trauma or cerebral disease and possible relatives with epilepsy.

All owners received a seizure calendar at the baseline visit and were instructed to record the date(s), time of day, duration and a detailed description of phenomenology if any new seizures should occur. Furthermore the owners were instructed to document seizure events on video if possible. Video recordings of seizures were used in the clinical evaluation.

For dogs with epilepsy where a need for treatment arose after inclusion, antiepileptic treatment was strongly recommended to the owner. The need for initiating antiepileptic treatment was decided on a case by case basis by the primary investigator (NF) in collaboration with an experienced veterinary neurologist/and human epileptologist (MB/AS).

### Longitudinal monitoring of dogs

After the clinical baseline visit the owners were contacted by telephone every eight weeks (+/- two weeks) until death or end of study and interviewed. A structured questionnaire (Questionnaire B) comprising four sections, was used for the telephone interviews:

1. Status (alive or dead): in case the dog had died since last investigator-owner contact, the time of death and cause of death were recorded.
2. Seizures since last follow-up: if new seizures had occurred, the owners were asked about date of seizure(s), seizure phenomenology, duration of seizures and possible occurrence of cluster seizures or/and status epilepticus.
3. Antiepileptic treatment: If receiving treatment, the owners were asked to state product, strength and dosage of the antiepileptic drug and to report occurrence of potential adverse effects. Furthermore owners were asked about possible owner initiated changes in drug regimens.
4. Occurrence of other disease or any medication given for other diseases than epilepsy since last investigator-owner contact.

In order to secure a consistent and structured evaluation of the dogs, all baseline investigations, interviews, subsequent telephone contacts and clinical investigations were performed for each single dog by the primary investigator (NF). The owners could furthermore contact the primary investigator at any time throughout the study.

Supplementary clinical control visits were conducted if any of the telephone interviews indicated new seizures (increase in seizure frequency) or any signs of progressive neurological disease or other diseases, which could promote seizures.

Dogs, in which a diagnosis of non-epileptic seizures of identified origin was established during the study, were subsequently referred to relevant hospital services for further monitoring and treatment.

### Statistical analysis

Descriptive analyses of qualitative data were performed for the following groups: epilepsy (idiopathic and structural epilepsy), non-epileptic seizures of identified origin, and suspected non-epileptic seizures of unidentified origin. Continuous data including age at seizure onset (months), age at inclusion (months), weight at inclusion, months of inclusion, age at death (months), numbers of seizures prior to inclusion and survival time were presented for dogs with idiopathic or structural epilepsy using median, range and quartiles (Q1-Q3). Median ages at seizure onset were compared using Mann–Whitney *U* test, a two-sided *P* value ≤ 0.05 was considered significant. For dogs with idiopathic epilepsy, the time interval (lengths of the interictal periods) from the first to the second epileptic seizure, from the second to the third epileptic seizure, and so on up to the fifth epileptic seizure were calculated and presented as median and quartiles (Q1-Q3). Dogs were included in this calculation up to the point where antiepileptic treatment was initiated. For untreated and treated dogs with idiopathic epilepsy, the number of dogs with a history of cluster seizures was compared using a Fischer exact test, a two-sided *P* value ≤ 0.05 was considered significant.

## Results

One hundred and twenty-three dogs were screened for study participation. Eight dogs failed inclusion due to the study exclusion criteria, leaving 115 dogs. Out of the 115 dogs, four dogs were excluded because the owners were unable to fully describe or document seizure events and five dogs were lost for longitudinal monitoring shortly after inclusion, leaving a final study population of 106 dogs which completed the study from inclusion to death or end of study. All dogs participating in the study were family dogs that lived indoors and were closely observed by their owners.

Seventy-nine dogs (74.5%) were diagnosed with epilepsy: 61 dogs (77.2%) with idiopathic epilepsy and 13 dogs (16.5%) with structural epilepsy. In five dogs (6.3%) structural epilepsy was strongly suspected. Thirteen dogs (12.3%) were categorized as having non-epileptic seizures of identified origin and ten dogs (9.4%) were categorized as having suspected non-epileptic seizures of unidentified origin. Four dogs (3.8%) experienced only a single seizure from seizure onset to the end of study, and no cause for the seizure event was identified. The distribution of dogs into the diagnostic categories appears from Fig. 1. Characteristics of the dogs in specific diagnostic categories are detailed in the following.

### Dogs with idiopathic epilepsy

Of the 61 dogs, 83.6% were purebred (distributed among 29 different breeds) and 16.4% were crossbreeds. Median age at seizure onset was 35 months (range: 5–114). Additional descriptive information of the dogs is presented in Table 1. At the time of study termination, 45 dogs were still alive, whereas 16 dogs had been euthanized. Ten dogs (62.5%) were euthanized due to epilepsy related causes, while in six dogs (37.5%) euthanasia was motivated by other non-neurological conditions. MRI was performed in 30 dogs (49%) of which 20 dogs also had a CSF analysis. All tests were normal. No clinical indications of other intra- or extracranial disease were identified for any of the 61 dogs from inclusion to death or end of study.

**Table 1:** Descriptive information for dogs with idiopathic or structural epilepsy

	Idiopathic		Structural	
	N	%	N	%
Status at study end				
Alive	45	74	4	31
Deceased	16	26	9	15
Gender				
Female	27	44	10	77
Entire	21	78	9	90
Neutered	6	22	1	10
Male	34	56	3	23
Entire	24	71	2	67
Neutered	10	29	1	33
Seizure type				
Focal	12	20	1	8
Focal evolving into generalized	34	56	9	69
Primary generalized	11	18	2	15
Unknown	4	7	1	8
Diagnostic work-up				
Magnetic resonance imaging	30	49	9	69
Computed tomography	0	0	4	31
Cerebrospinal fluid analysis	20	33	6	46
Post mortem histopathology	1	2	6	46
Seizure severity				
Cluster seizures (prior to inclusion)	18	30	12	92
Status epilepticus (prior to inclusion)	2	3	0	0
Median age at seizure onset (months)^a^	35 (range: 5–114)		59 (range 3–144)	
Median age at inclusion (months)	43 (range: 13–119)		48 (range: 3–146)	
Median months of inclusion	28 (range: 1–42)		1 (range: 0–38)	
Median weight at inclusion (kg)^b^	23.9 (range: 2–71)		8.9 (range: 2.2–32)	
Survival				
Survival time (months)	23 (range: 3–65)		2 (range: 0–49)	

^a^Seven dogs with idiopathic epilepsy and two dogs with structural epilepsy excluded, as exact date of onset was unknown

^b^Three dogs with idiopathic epilepsy excluded from calculation, as weight at onset was unknown

Twelve dogs (19.7%) experienced focal seizures alone, whereas 34 dogs (55.7%) experienced focal seizures evolving to become generalized, and 11 dogs (18.0%) had primary generalized seizures. In four dogs (6.6%) it was not possible to classify seizure type because owners were unable to describe if convulsions were preceded by other events e.g. cases where the owner would wake up at night finding the dog seizing. In 53 dogs (86.9%) the owners reported a postictal phase lasting more than two minutes.

The median number of epileptic seizures prior to inclusion was three (Q1-Q3: 2–4, range 1–11) and the median time of inclusion in the study was 28 months (Q1-Q3: 23–32, range 3–42). Only four dogs were included in the study for less than 12 months due to euthanasia; three of these dogs were euthanized because of severe epilepsy and poor seizure control and one dog was euthanized because of severe elbow dysplasia. MRI and CSF analysis were performed in three and two of the four dogs, respectively, with normal results.

The median time interval between epileptic seizures from the first to the second seizure, the second to the third seizure and so on up to the fifth seizure was estimated in up to 48 dogs with idiopathic epilepsy (80%) and ranged from a median of 50.5–72 days (Table 2). Only untreated dogs in which the exact date of first seizure was known were included in the calculations and as soon a dog started treatment it was excluded from calculations. Thirteen dogs were excluded from calculation, as the exact date of first seizure was uncertain. No shortening of the median time interval between epileptic seizures (from the first to fifth seizure) and thus no signs of an accelerating course of epilepsy was observed. A second epileptic seizure occurred within one month of the first in 14 dogs (29.2%), within three months in additional 15 dogs (29 dogs in total) (60.4%) and within one year in additional 15 dogs (44 dogs in total) (92.2%). In four dogs a second seizure occurred between one and two years after the first seizure.

**Table 2:** Median time interval from the first to the fifth seizure in untreated dogs with idiopathic epilepsy

	Number of dogs	Median time between seizures (days)	Q1-Q3
From 1st to 2nd^a^	48	72	27.75–156.25
From 2nd to 3rd^b^	46	50.5	20.5–120.5
From 3rd to 4th^c^	42	52	29.5–123,75
From 4th to 5th^d^	35	55	15.5–175

^a^Thirteen dogs excluded from calculation, as there was uncertainty of the exact date of first seizure

^b^One dog excluded, as it did not have a third seizure and one dog excluded as treatment was initiated

^c^Three dogs excluded, as they did not experience a fourth seizure and one dog excluded as treatment was initiated

^d^Four dogs excluded, as they did not experience a fifth seizure and three dogs excluded as treatment was initiated

**Fig. 2:**
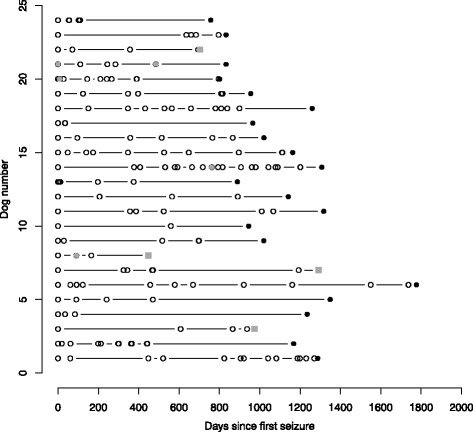
Seizure recurrence in 24 untreated dogs with idiopathic epilepsy. Open circles = seizure. *Black* circles = end of study. *Gray* circles = cluster seizure. *Gray* squares = Death/euthanasia

Twenty-six dogs remained untreated (43%) from study start to the end of study. Seizure recurrence (from seizure onset to end of study) is illustrated for 24 out of 26 untreated dogs in Fig. 2 (two dogs were excluded from calculation, as the owner forgot to register a few seizures in the seizure calendar). As it appears from Fig. 2 the seizure patterns were characterized by being very individual and fluctuating. Some dogs such as dog 1 and 14 experienced a progressive course of the disease, while other dogs e.g. dog 9 and 11 experienced prolonged periods of seizure freedom. When looking at the lengths of all interictal periods in the 24 dogs, the longest interictal period was a median of 408.5 days (Q1-Q3: 334.25–639, range: 238–1151), while the shortest interictal period was a median of 20 days (Q1-Q3: 3–71, range: 1–559).

Thirty-five dogs (57%) started antiepileptic treatment at some point during the study. When comparing untreated and treated dogs, significantly (*P* = 0.014) more dogs in the treatment group had a history of cluster seizures compared to the dogs, which remained untreated reflecting that the primary investigator advocated for early treatment in dogs with cluster seizures. However the owners’ opinions also influenced the decision of treatment as it appears from Fig. 2.

Eleven out of the thirty-five treated dogs participated in a concurrent clinical trial where dogs were randomized to mono-therapy with either phenobarbital (*n* = 6) or levetiracetam (*n* = 5) [20]. Of these, nine out of eleven dogs experienced five or more seizures before intiating treatment. For the remaining dogs mono-therapy was instituted with phenobarbital (*n* = 22) or with imepitoin (*n* = 2), which was approved in Europe during the study. Against the primary investigators recommendations, some owners declined starting antiepileptic treatment in their dog, explaining why some dogs e.g. dog 1, 6, 14 and 15 (Fig. 2) did not receive therapy despite a high seizure frequency and in some cases cluster seizures.

### Dogs with structural epilepsy

Out of 13 dogs with structural epilepsy, 92.3% were purebreds (distributed among 11 different breeds) and 7.7% were crossbreeds. Median age at seizure onset was 59 months (range: 3–144). Additional descriptive information on the dogs appears from Table 1. These cases were characterized by anamnestic information or neurological findings (alone or in combination) indicating structural brain pathology. Ten dogs (76.9%) experienced focal seizures, which for nine dogs evolved to become generalized and two dogs (15.4%) experienced primary generalized seizures. In one dog (7.7%) it was not possible to classify seizure type. Owners reported a postictal phase lasting more than two minutes in all 13 dogs (100%). In all cases the diagnosis was confirmed with MRI, CSF analysis and/or post mortem examination. Six dogs (46.1%) were diagnosed with inflammatory disease, two dogs (15.4%) with neoplasia, two dogs (15.4%) with malformation (hydrocephalus), one with spongiform leucoencephalopathy (7.7%) and two dogs (15.4%) with cerebral hemorrhagic lesions due to Angiostrongylus Vasorum induced coagulopathy. In the latter two dogs, the onset of seizures occurred five months and four years respectively after the primary incident, presumably due to the post hemorrhagic chronic lesion.

At the time of study termination, four dogs (30.8%) were still alive while nine dogs (69.2%) had been euthanized due to the primary brain disease and progression of epileptic seizures. Median survival time after diagnosis was 2 months, range: 0–49 months.

No statistically significant difference in the median age at seizure onset was found between dogs with idiopathic epilepsy and dogs with structural epilepsy (*P* = 0.33).

### Dogs with suspected structural epilepsy

Five dogs, of which three were females (all intact) and two were males (both intact), presented with a history indicating structural brain pathology or an abnormal neurological examination, which indicated structural epilepsy. This could, however, not be confirmed because the owners declined advanced neuro-diagnostics such as MRI, CSF analysis and post mortem examination. There was no indication of other diseases known to mimic epilepsy. At the end of the study two dogs were still alive while three dogs had been euthanized. The two dogs, that were still alive at the end of the study, were included in the study for 24 and 29 months, respectively. No progression in the number of seizures was observed during the course of the study in any of the two dogs.

### Dogs with non-epileptic seizures of identified origin

Thirteen dogs, eight females (six intact, two neutered) and five males (four intact, one neutered) were included in this group. The causes of seizures were confirmed as cardiovascular syncope (*n* = 5), brachycephalic syndrome (*n* = 2), Bulldog idiopathic head tremor (*n* = 2), immune mediated hemolytic anemia (*n* = 1), portosystemic shunt (*n* = 1), nsulinoma (*n* = 1) and Border collie collapse (*n* = 1). For nine dogs (69.2%) a triggering factor (coughing, play, excitement or high activity) preceded seizure events. Owners reported a post seizure recovery phase lasting more than two minutes in eight dogs (61.5%), while five dogs (38.5%) had a postictal phase lasting less than two minutes. The different diagnoses were equally distributed between dogs experiencing a short and a long recovery phase after seizures.

The median age at seizure onset in this group was 43 months (Q1-Q3: 15.25–79.75, range: 6–136; three dogs were excluded from calculation, as the exact date of seizure onset was uncertain).

No significant difference was found in the median age at seizure onset between dogs with non-epileptic seizures of identified origin and the two epilepsy types, idiopathic epilepsy (*P* = 0.98) and structural epilepsy (*P* = 0.79), respectively.

### Dogs with suspected non-epileptic seizures of unidentified origin

Ten dogs, seven females (six intact and one neutered) and three males (all intact), were included in this group All dogs had a normal clinical and neurological examination and had CBC, biochemistry and thyroid profiles within normal ranges. The median age at seizure onset was 43 months (Q1-Q3: 3–46, range: 3–104). One dog was excluded from calculation, as the exact date of seizure onset was uncertain.

Two dogs were suspected of cardiovascular disease, as seizures only occurred in relation to high activity/acute stressful situations and the ictal phase lasted less than a minute with no postictal phase. In both cases the owners declined Holter monitoring, radiographs, fluoroscopy and other suggested diagnostic tests. MRI and CSF analysis were not performed. One dog was suspected of a sleeping disorder. The owner documented an average of 10 seizure events occurring during sleep every night. The owner declined MRI and CSF analysis, and exploring antiepileptic treatment as an alternative diagnostic test. A paroxysmal movement disorder was suspected in three dogs in which seizure events were characterized by episodic longer lasting (up to hours) involuntary contractions of skeletal muscles (front leg in 1 dog, hind limb in 1 dog, and involving several groups of skeletal muscles in 1 dog) with absence of other clinical signs or neurological signs and a normal neurological examination. These dogs appeared mentally bright and alert during seizures. One of the dogs had MRI and CSF performed with normal results (the dog with generalized involuntary muscle contractions). Besides that, all owners declined further diagnostics e.g. muscle biopsy. In the last four dogs the seizures were classified as being of unknown origin due to an atypical nature of the seizures and lack of further diagnostic work-up.

At study termination eight dogs were still alive, while two dogs had been euthanized due to other reasons than seizures.

## Discussion

The longitudinal study design of this observational study allowed us to provide prospectively collected longitudinal descriptive information about dogs with newly diagnosed epilepsy and its suspected and confirmed differential diagnoses, which has not been done before. We found that epilepsy was by far the most frequent diagnosis in dogs presenting with a history of new-onset seizures (excluding SE), which is important information for first-line practitioners. The considerable individual variation in epileptic seizure frequency and epileptic seizure density in the group of dogs with idiopathic epilepsy, which remained untreated throughout the study certainly illustrated the unpredictable nature of epilepsy and exemplifies the difficulties associated with predicting the course of the disease and also underlines the fact that some dogs might actually not need treatment.

Dogs with structural epilepsy were characterized by anamnestic information or neurological deficits or both at presentation indicating cerebral dysfunction, and this represent an important clinical diagnostic marker. Focal seizures evolving to become generalized were the most common types of epileptic seizures in dogs diagnosed with idiopathic and structural epilepsy, which is in accordance with was has been reported previously [21–24]. The distribution of focal onset seizures and generalized seizures was similar between dogs with idiopathic epilepsy and dogs with structural epilepsy, and thus we did not find any indication that the seizure type can be a helpful clinical marker of specific epilepsy etiology. The majority of dogs with epilepsy (idiopathic and structural) experienced a postictal phase lasting more than two minutes, which was also the case for approximately 60% of the dogs with non-epileptic seizures of identified origin. Based on these findings, a postictal phase lasting more than two minutes does not seem to advocate more for epilepsy than for a non-epileptic seizure condition. Dogs with non-epileptic seizures of identified origin were predominantly characterized by a history where seizure events were preceded by a specific immediate triggering factor such as e.g. physical activity or excitement, which might represent a clinical diagnostic marker.

With respect to age at seizure onset, no significant difference was found in the median age at seizure onset when comparing dogs with idiopathic and structural epilepsy and epilepsy and non-epileptic seizures of identified origin. Our results indicate that age at seizure onset might not be a good clinical marker when seeking to differ between epilepsy types (idiopathic and structural) or between seizures of epileptic or non-epileptic origin and thus contradicts previous suggestions that age at seizure onset could be an indicator of etiology [1, 3, 25]. We however acknowledge that the number of dogs diagnosed with structural epilepsy and non-epileptic seizures of identified origin was relatively low, which might have affected the results. It would be interesting to investigate the findings of the present study in a future large-scale epidemiological study.

In humans, only around 40% experience recurrence of seizures after a first unprovoked seizure [5, 6]. This was not the case in the present study where only four dogs (3.8%) experienced a single seizure and no seizure recurrence. We, however, believe that a number of dogs with only one seizure are never registered, as owners might await more seizures before seeking veterinary advice. It is therefore highly possible that we have missed to include such cases in our study and thus this result is probably underestimated.

In more than half of the dogs diagnosed with idiopathic epilepsy a second epileptic seizure occurred within three months after the first epileptic seizure, and in 92% of the dogs within one year. These findings are in accordance with a retrospective study of the course of untreated epilepsy in people with tonic-clonic seizures [26].

In humans as well as dogs it was for many years claimed that *“seizures beget seizures”* [27]. Evidence-based clinical information, which can support this hypothesis is sparse, and at least in humans immediate initiation of treatment does not seem to influence long-term outcome [5, 6]. This is in accordance with the present study where we could not demonstrate an accelerating course of idiopathic epilepsy when comparing the median time interval between the first to the fifth epileptic seizure in untreated dogs.

Seizure recurrence has previously been investigated in one veterinary study, which found that the probability of primary epilepsy (idiopathic epilepsy) was significantly higher than a diagnosis of secondary epilepsy (structural epilepsy) or reactive seizures, if the time between the first and the second seizure exceeded four weeks [1]. The present study confirmed a median time interval between the first and the second epileptic seizure of more than four weeks (72 days) in dogs with idiopathic epilepsy.

The recently published consensus proposal from the IVETF and the 2015 ACVIM Small Animal Consensus Statement on seizure management in dogs recommends initiation of long-term antiepileptic treatment when two or more epileptic seizures have been observed within a six month period [28, 29]. Our findings in untreated dogs with idiopathic epilepsy, which were long-term monitored, might however suggest that there is a potential risk of unnecessary treatment when antiepileptic medication is initiated routinely after a second seizure occurring within six months. As many idiopathic epilepsies in dogs (as opposed to humans) are familial (breed related) and of a suspected genetic origin [30] the decision of when to initiate antiepileptic treatment in dogs with idiopathic epilepsy might need to be individualized. This decision seems to be best rooted in current knowledge about the characteristics of the course of epilepsy in the breed in question (epilepsy phenotype), including epileptic seizure phenomenology, epileptic seizure frequency and density, the risk of cluster seizures and status epilepticus, severity of post-ictal events and response to treatment.

In nearly 60% of the dogs with idiopathic epilepsy a need for antiepileptic treatment was identified at some point during the study. In the present study the recommendation on when to initiate antiepileptic treatment was made on a case by case basis by the primary investigator in collaboration with an experienced veterinary neurologist/and human epileptologist. For all dogs with cluster seizures therapy was strongly advised. For some dogs it was necessary to institute treatment during the study, whereas for the 26 dogs which remained untreated throughout the study, we had the opportunity to follow seizure recurrence over a long period of time. We could have chosen to set a fixed number of seizures to have occurred as a starting point for initiating treatment, but we deliberately chose not to do so in order to reflect “real life” with epilepsy patients as much as possible. In daily life with epilepsy patients any initiation of treatment is based on a combination of the clinician’s recommendations and the owner’s motivation - which we were also able to document in the present study. Our approach may have its limitations, but we still find that our study provide important informative observational data.

The fact that owners’ opinions indeed influence the decision of when to start treatment was highlighted by the present study (reflected in Fig. 2). This has also been reported by other investigators [31–33]. Our study also depicted that some owners will decline treatment against the professional advice given by the veterinarian working to improve the quality of life for the affected animal.

Eleven dogs with idiopathic epilepsy participated in a concurrent clinical treatment trial [20], which potentially could have been a confounding factor with respect to the calculations concerned with investigating the risk of an accelerating course of epilepsy (from the first to the fifth epileptic seizure). The risk of confounding is however considered minor, as the majority of these dogs (*n* = 9) experienced 5 or more seizures before initiating treatment.

A history of cluster seizures was significantly present in the treated dogs compared to those that remained untreated. This is however not surprising, as prompt initiation of antiepileptic treatment as a rule is recommended for dogs experiencing cluster seizures [28, 29, 34]. This was also the case in the present study.

Twelve percent of the included dogs in our study were diagnosed with non-epileptic seizures of identified origin. This is in accordance with two previous studies, which found an underlying metabolic or toxic cause in 10% and 11% of dogs suffering from seizures [1, 2].

In two dogs, the cause of epileptic seizures was evaluated to be a post hemorrhagic cerebral lesion evidenced by MRI and caused by a previous infection with Angiostrongylus vasorum. Signs of neurological disease including epileptic seizures have previously been described in dogs in the acute states of Angiostrongylus vasorum infection [35, 36]. Late onset of epileptic seizures secondary to a hemorrhagic incident is not well documented in dogs but has been reported in humans [37, 38]. One human study reported a median delay of seizures of nine months (Q1-Q3: 3–23 months) from the hemorrhagic incident [38].

A common bias when investigating epileptic dogs in a referral hospital setting is that such dogs cases are referrals which are commonly dominated by severe epilepsy characterized by a high seizure frequency and in some cases pharmacoresistance, and thereby they might not truly reflect a normally distributed population of epileptic dogs. The dogs participating in the present study were recruited after a median of three seizures and were untreated at inclusion, which would also be a common scenario in first-line practice. Thus we consider bias with regard to an overrepresentation of severe epilepsy cases to be minor.

We fully acknowledge that certain limitations are associated with a longitudinal observational study design as the present, e.g. the fact that investigators must respect when owners decline some diagnostic tests in order to comply with the overall ethical rules associated with studies including companion dogs. This is also the case in first-line clinics. MRI could therefore not be performed in all dogs diagnosed with idiopathic epilepsy, why it cannot be excluded that structural epilepsy cases e.g. occult developmental cerebral anomalies, slower growing benign tumors, and silent vascular insults were included in the group of dogs with idiopathic epilepsy. However, diagnostic imaging was performed and appeared normal in 49% of the dogs diagnosed with idiopathic epilepsy, and furthermore the majority of dogs were followed for a minimum of one year where they did not develop any signs of neurological disease. This longitudinal monitoring served to support the validity of the diagnoses. Therefore we consider the likelihood of a progressive intracranial lesion to be limited.

The benefits of using a prospective observational longitudinal study design (implicating pre-scheduled regular investigator-owner contacts) is obviously the advantage of being able to monitor clinical progression/regression of disease and report data which is prospectively collected over a longer period of time. This is not a possibility with retrospective studies. Our study thus had the design to illustrate the distribution of non-epileptic and epileptic causes for seizures in dogs with new-onset seizures, and furthermore contributed with a peace of the puzzle forming the so-called “natural history of epilepsy” [7].

## Conclusions

The present study demonstrated that epilepsy is the most probable diagnosis in dogs presenting with new-onset seizures.. The consequences of a diagnosis of idiopathic epilepsy are very individual and some dogs may not need antiepileptic treatment. This information can be useful for clinicians when advising owners of dogs with new-onset seizures about which diagnostic approach to take and what to expect with regard to prognosis. Our results might also be of interest for future epilepsy treatment recommendations.

## Abbreviations

ACVIM: American College of Veterinary Internal Medicine
CBC: Complete blood count
CSF: Cerebrospinal fluid
CT: Computer tomography
fT4: Free thyroxin
IVETF: International Veterinary Epilepsy Task Force
MRI: Magnetic resonance imaging
POMR: Problem oriented medical record
SE: Status epilepticus
TSH: Thyroid stimulating hormone
TT4: Total thyroxin
